# ORFcor: Identifying and Accommodating ORF Prediction Inconsistencies for Phylogenetic Analysis

**DOI:** 10.1371/journal.pone.0058387

**Published:** 2013-03-06

**Authors:** Jonathan L. Klassen, Cameron R. Currie

**Affiliations:** 1 Department of Bacteriology, University of Wisconsin-Madison, Madison, Wisconsin, United States of America; 2 DOE Great Lakes Bioenergy Research Center, University of Wisconsin-Madison, Madison, Wisconsin, United States of America; J. Craig Venter Institute, United States of America

## Abstract

The high-throughput annotation of open reading frames (ORFs) required by modern genome sequencing projects necessitates computational protocols that sometimes annotate orthologous ORFs inconsistently. Such inconsistencies hinder comparative analyses by non-uniformly extending or truncating 5′ and/or 3′ sequence ends, causing ORFs that are in fact identical to artificially diverge. Whereas strategies exist to correct such inconsistencies during whole-genome annotation, equivalent software designed to correct subsets of these data without genome reannotation is lacking. We therefore developed ORFcor, which corrects annotation inconsistencies using consensus start and stop positions derived from sets of closely related orthologs. ORFcor corrects inconsistent ORF annotations in diverse test datasets with specificities and sensitivities approaching 100% when sufficiently related orthologs (e.g., from the same taxonomic family) are available for comparison. The ORFcor package is implemented in Perl, multithreaded to handle large datasets, includes related scripts to facilitate high-throughput phylogenomic analyses, and is freely available at www.currielab.wisc.edu/downloads.html.

## Introduction

Recent technical advances have promoted the proliferation of genome sequencing projects, leading to the accumulation of extensive genome-scale sequence data in public databases. This has in turn facilitated routine, large scale comparative analyses of functional and taxonomic diversity, i.e., "phylogenomics" [1], [2]. Most phylogenomic approaches require comparisons between genes or proteins, e.g., to determine homology, identify orthologous, paralogous and xenologous relationships, and conduct phylogenetic analysis. Such analyses assume that their input data are directly comparable, i.e., a sequence that is truly 100% identical in two genomes will exist in exactly identical copies in each genome. However, the computational methods typically used to annotate open reading frames (ORFs) in these data are never 100% accurate [3], [4], [5], differ between groups and over time, and are typically not validated experimentally due to limited resources. Whereas orthologous ORFs may truly differ in structure (e.g., due to multiple unique start or stop sites, programmed frameshifts, or pseudogenization [6]), differentiating such genuine variation from sequencing or annotation errors is difficult without experimental validation. It may also be desirable from a phylogenomic perspective to ignore such differences, e.g., due to the reversibility of programmed frameshifts or the exclusion of the longer of two genuine start sites. Incomplete ORFs may also result from genome fragmentation in draft-quality and metagenomic data, which both omits the missing sequence region and increases the difficulty of accurate gene prediction [7], [8], [9]. Low-quality genomes may also contain chimeras resulting from the erroneous merging of disparate sequences into a single contig, a relatively common occurrence even when using state-of-the-art genome assembly methods [10], [11]. Chimeric ORFs may also result from the inappropriate merging of two different read frames, either erroneously due to sequencing errors or genuinely because of a frame shift mutation. Such chimeric ORFs will therefore be erroneously truncated and concatenated to some unrelated sequence. The result of any of these inconsistencies is that two truly identical sequences will artificially differ due to ORF truncation, extension, and/or the incorrect incorporation of sequence not belonging to that ORF, thereby potentially confounding further analysis.

Inconsistent computational ORF prediction is the best studied of the above biases. Algorithms differ considerably in the ORF sets that they predict [3], [4], [5] and in their ability to differentiate between coding and non-coding sequences [12], [13]. For example, Hyatt et al. [3] reported errors in 3.3–13.1% of computationally predicted ORFs compared to experimentally derived values, depending on the ORF prediction algorithm used and taxon examined. The start sites predicted using different algorithms are especially known to vary [14]. Organisms having high %G+C content are particularly susceptible to ORF prediction errors [12], [15], especially due to increased incidence of the alternative start codon GTG in such genomes. Indeed, underannotation of the ATG start codon in favor of GTG has been noted as a pervasive problem [16], [17]. These inconsistencies occur frequently in publicly available databases such as Refseq [16], [17], [18], and are often not conserved between genomes. Highlighting the latter, Dunbar et al. [18] identified inconsistencies in 53% of ortholog sets constructed from the GenBank annotations of five *Burkholderia* genomes. ORF prediction inconsistencies therefore have the potential to significantly affect comparative genomics.

Several groups have improved ORF prediction consistency during genome annotation (i.e., genomewide ORF prediction) by combining multiple gene prediction algorithms and leveraging the structure of publicly-available ORF predictions [19], [20], [21], [22], and excluding unlikely structures such as extensive ORF overlaps [23], frameshifts resulting from sequencing errors [6], and ORFs with otherwise aberrant sequence signatures [13]. However, to our knowledge, software does not exist to correct inconsistencies outside of a genome annotation context, e.g., for phylogenetic analysis of selected ortholog families. We therefore developed ORFcor to detect and correct three ORF prediction inconsistency types (ORF overextension, truncation and chimerism) without genome reannotation by leveraging the structures of closely related orthologs. ORFcor uses the consensus approach of Wall et al. [22] to detect and correct inconsistent ORF predictions in public data. Because such inconsistencies are typically distributed widely in bacterial genomes without specific localization to particularly problematic genomic regions [18], inconsistent ORFs will comprise a minority of most ortholog sets. In addition to working outside of a genome annotation context, ORFcor differs from previous approaches by using reference sequence sets having high similarity to query sequences (e.g., vs. using BLASTx vs. nr as in GenePRIMP [21]) and using protein sequences to more robustly compare divergent orthologs (e.g., vs. using nucleotide sequences as in GMV [22]). Provided that truncated ORFs do not comprise the majority of the ortholog set, ORFs from draft-quality genomes, metagenomics and PCR amplicon sequencing can also be corrected using this method. Finally, we further integrated the ORFcor algorithm within a modular pipeline for high-throughput phylogenomic analysis to facilitate its routine application and customization using other methods for ortholog detection and correction.

## Materials and Methods

### ORFcor Algorithm Overview

ORFcor requires sets of orthologous protein or nucleotide sequences as input. Any method to generate such ortholog sets is compatible with ORFcor, so long as each set exists as a separate fasta formatted file. Nucleotide sequences are translated to protein sequences, analyzed normally by ORFcor, and the resulting corrections back-translated to nucleotide sequences by replacing "X"s with equivalent strings of "N"s. Nucleotide sequences must be free of indels to ensure their proper translation. Translating nucleotides to their corresponding protein sequences maintains proper reading frames and increases the similarity between sequences by effectively only considering non-synonymous sequence differences, an important criterion for maximizing the accuracy of ORFcor (see below). Nucleotide sequences are not compatible with the other steps of the ORFcor pipeline.

**Figure 1:**
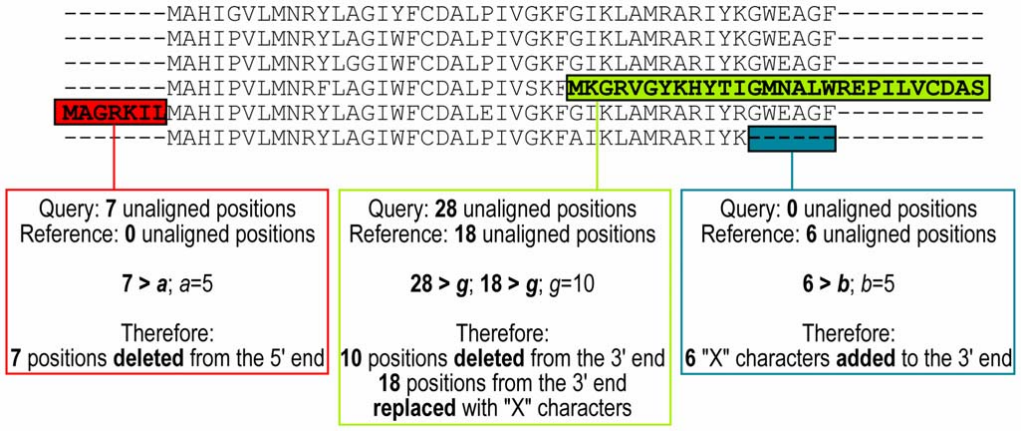
Illustrative examples of the ORFcor approach. A sequence alignment is given where one sequence is overextended (red box), one is chimeric (green box) and one is truncated (blue box). For each altered sequence, the consensus unaligned sequence positions for both the query and reference are indicated, compared with the relevant (non-default) parameters, and the resulting alterations to the sequences indicated.

Within these ortholog sets, each sequence (“query”) is aligned to all others in its “reference” ortholog set using BLASTp in the BLAST+ package [24] using default parameters except "-comp_based_stats F" and "-evalue" and "-max_target_seqs" as defined by ORFcor parameters "-e" and "-j", respectively (1e-5 and 5 by default, respectively). By default, ≥5 reference orthologs exceeding an identity threshold value *d* (the fraction of identities shared between both sequences in the aligned region; by default 0.9) are required to attempt correction, yielding a theoretical false detection rate <2% [22]. The extent to which each query and reference are misaligned at both 5′ and 3′ sequence ends is recorded until the number of comparisons exceeds a threshold or the number of stored BLAST hits (*k* and *l*, respectively, both by default 1000). All references 100% identical to the query are considered (≤ *l*) to avoid bias towards overextended references, which will have higher BLAST scores versus the query compared to shorter, but otherwise identical reference sequences. Consensus start and stop sequences are determined as the number of unaligned query and reference amino acids (AA) at their 5′ and 3′ sequence ends calculated for all query-reference comparisons. Potential consensus start and stop positions are considered if they agree in ≥33% of the query–reference comparisons for each query sequence, defaulting to the longest consensus if more than one are found. If the consensus number of unaligned AA at the 5′ or 3′ ends of both the query and reference exceed a threshold (5': *f*, by default 10 AA; 3': *g*, by default 30 AA) the query is considered chimeric, truncated to the consensus query alignment start or end position and (consensus reference alignment start or end position)−1 “X” characters are added. Alternatively, if the consensus number of unaligned query AA exceeds a threshold (5': *a*, by default 5 AA; 3': *b*, by default 20 AA), the query is considered truncated and (consensus reference alignment start or end position)−1 “X” characters are added to that query sequence end to denote missing data. Finally, if the consensus number of unaligned reference AA ≥ *a* or *b*, the query is considered inappropriately extended and truncated to the consensus reference alignment start or end position. An example of each error type is illustrated in Figure 1. Because alignments are calculated sequentially for each query sequence, datasets comprising two equally common start or stop sites may trigger both trimming and overextension of opposing halves of the same dataset. This difficulty is overcome by only allowing extensions for ortholog sets where ≥40% of the sequence ends are proposed for trimming and a second ≥40% are proposed for overextension, calculated using only query sequences that can be corrected according to the ORFcor input parameters.

### Test Datasets and Validation

To examine the performance of ORFcor, we obtained the predicted proteomes for 1519 complete genomes available in the NCBI FTP folder “genomes/Bacteria/" as of January 4, 2012. The COG seed sequences for each of 31 ortholog families conserved in nearly all bacteria with minimal horizontal gene transfer (and thereby most suitable for phylogenomic analysis) [25] were downloaded from the Conserved Domain Database [26], aligned using MUSCLE v3.70 according to default parameters [27], and used as input for HMMER v3.0 [28] to create hidden Markov models (HMMs) for each family. Each proteome was queried using these HMMs with expectation cutoffs manually set for each HMM, typically 1e^−20^ lower than the expectation value at which the correct orthologs were recovered from endosymbiont genomes for which small population sizes have caused extensive sequence divergence [29]. Using such stringent parameters will avoid most paralogous sequences but will also omit some genuine orthologs poorly matching the HMMs, especially if these orthologs are truncated, e.g., due to draft-quality genome sequencing. We provide these HMMs and multithreaded scripts to query multiple genomes using HMMER as part of the ORFcor package, although any other method for generating sets of orthologs can also be used; for recent reviews of such methods see [30], [31].

Unfortunately, no set of closely-related genomes exists having ORFs annotated with 100% consistency and therefore suitable to parameterize the ORFcor algorithm. To approximate such a dataset, we selected γ-proteobacterial genomes from the dataset described above belonging to genera containing >5 strains (excluding *Buchnera*; see below), identified 31 ortholog sets as described above, aligned each using MUSCLE, visually identified ORF annotation inconsistencies, and corrected them according to their closest homologs in this dataset. This reference dataset contained 5438 protein sequences representing 176 strains in 13 genera (range: 6–30 strains per genus). To simulate data containing annotation inconsistencies, these corrected sequences were artificially extended, truncated, or had their sequence ends replaced by divergent sequences to create chimeras (see ORFcor package for datasets and scripts to replicate these experiments). For each parameter setting, only sequences having ≥5 unmodified orthologs within the chosen parameter settings were altered. The length of the added or deleted sequences were Poisson distributed above a set threshold, and added sequences were derived from COG families unrelated to the analyzed orthologs. Multiple test datasets were created using various combinations of error frequencies and lengths, with representative results shown in Table 1 and the complete dataset in File S1. The simulated error frequencies were substantially higher than those observed during preliminary experiments, and using lower frequencies only increased the performance of the algorithm. Fifty replicate test datasets were created for each parameter combination.

**Table 1:** Performance of ORFcor run on simulated inconsistency-containing data in comparison to known values using the parameters: *a = *5; *b* = 10; *d* = 0.75 or 0.90; *f = *10; *g = *30; *l = k = *1000.

	*d* = 0.75		*d* = 0.90	
	Test Dataset #1	Test Dataset #2	Test Dataset #1	Test Dataset #2
*5′ Chimeras*	*Length≥10 AA*	*Length≥20 AA*	*Length≥10 AA*	*Length≥20 AA*
S_p_	100.00%	99.99%	100.00%	100.00%
S_n_	67.33%	92.00%	64.00%	88.33%
Mean 100% accurate corrections^a^	83.66±1.85%	55.43±1.19%	86.98±1.56%	62.26±1.53%
Mean deviation from perfect correction^b^	1.18±0.46 AA	3.76±2.92 AA	1.04±0.20 AA	3.29±2.79 AA
*3′ Chimeras*	*Length≥30 AA*	*Length≥40 AA*	*Length≥30 AA*	*Length≥40 AA*
S_p_	100.00%	100.00%	100.00%	100.00%
S_n_	65.00%	87.33%	62.67%	85.33%
Mean 100% accurate corrections	82.05±1.56%	53.82±1.52%	85.11±1.78%	63.28±1.53%
Mean deviation from perfect correction	1.34±0.68 AA	3.06±2.24 AA	1.14±0.36 AA	3.29±2.64 AA
*5′ Overextensions*	*Length≥5 AA*	*Length≥10 AA*	*Length≥5 AA*	*Length≥10 AA*
S_p_	99.72%	99.72%	99.64%	99.66%
S_n_	99.82%	99.82%	98.58%	98.52%
Mean 100% accurate corrections	98.65±1.91%	98.75±2.13%	99.52±3.23%	99.49±3.23%
Mean deviation from perfect correction	9.37±22.49 AA	9.14±20.97 AA	10.56±17.26 AA	9.51±16.79 AA
*3′ Overextensions*	*Length≥20 AA*	*Length≥25 AA*	*Length≥20 AA*	*Length≥25 AA*
S_p_	100.00%	100.00%	100.00%	100.00%
S_n_	98.15%	100.00%	97.78%	98.80%
Mean 100% accurate corrections	95.59±2.09%	94.80±1.75%	96.99±2.04%	96.61±1.86%
Mean deviation from perfect correction	2.10±2.60 AA	2.01±2.44 AA	1.00±0.00 AA	1.21±1.00 AA
*5′ Truncations*	*Length≥5 AA*	*Length≥10 AA*	*Length≥5 AA*	*Length≥10 AA*
S_p_	99.80%	99.81%	99.99%	99.99%
S_n_	98.97%	99.41%	98.07%	97.99%
Mean 100% accurate corrections	93.14±5.10%	90.65±4.58%	95.91±5.05%	94.38±4.95%
Mean deviation from perfect correction	2.61±12.08 AA	1.64±1.52 AA	2.40±3.27 AA	1.80±1.33 AA
*3′ Truncations*	*Length≥20 AA*	*Length≥25 AA*	*Length≥20 AA*	*Length≥25 AA*
S_p_	100.00%	100.00%	100.00%	100.00%
S_n_	97.45%	99.49%	97.56%	97.78%
Mean 100% accurate corrections	88.80±2.53%	86.77±2.64%	94.51±2.42%	92.51±2.57%
Mean deviation from perfect correction	1.67±1.50 AA	2.12±2.15 AA	1.21±1.59 AA	0.97±1.34 AA

The lengths of the simulated errors are indicated for each error type; test dataset #1 represents the shortest possible errors detectable using the tested ORFcor parameters. Errors were introduced into test sequences at rates: 5′ overextensions and truncations - 5%; 3′ overextensions and truncations - 1%; 5′ and 3′ chimeras - 0.1%. See File S1 for the complete simulation results.

a Mean 100% accurate correction values and their standard deviations were derived from all true-positive values, averaged over the 50 replicates run for each parameter set.

b Mean deviation from perfect correction is derived from true-positive values that are not 100% accurate.

ORFcor was run on each set of replicate test datasets using a range of parameters. For each test set, the number of true and false positive corrections (TP and FP hereafter) and the number of true and false sequences left uncorrected (i.e., negative corrections; TN and FN hereafter) were recorded for each corrected dataset as compared to the inconsistencies added during test dataset construction. The sensitivity, specificity, precision and F-score for each inconsistency type were calculated for the union of all TP, FP, TN and FN values across all 50 replicate test datasets for each parameter value as: sensitivity (S_n_) = TP/(TP+FN) * 100%; specificity (S_p_) = TN/(FP+TN) * 100%; precision = TP/(TP+FP) * 100; and F-score = 2 * (precision * S_n_)/(precision+S_n_) * 100. The degree to which the positions of the introduced modifications corresponded to those of the actual inconsistencies introduced into the test datasets was also recorded.

### Pipeline Overview

In addition to the ORFcor algorithm itself, we provide a fully integrated pipeline for high -throughput phylogenomic analysis. All steps of this pipeline except the last are multithreaded to accommodate the often computationally taxing size of modern datasets, and constructed modularly to facilitate customization. Although principally directed towards microbial protein sequences, it can easily be adapted to other taxa and data types (although because it does not consider indel sequences, it is not suitable for use with raw eukaryotic DNA sequences). The steps of the ORFcor pipeline are:

1. HMMER_model_maker.pl: A multithreaded wrapper script to construct a set of HMMs using HMMER3 [28].
2. Mult_hmmscan.pl: A multithreaded wrapper script to query target genomes using the HMMs generated in step 1 (or via other equivalent methods).
3. ORF_cor.pl: A multithreaded implementation of the ORFcor algorithm, which corrects ortholog sets generated using steps 1 and 2 (or via other equivalent methods).
4. Mult_MUSCLE.pl: A multithreaded wrapper script to make multiple sequence alignments of each corrected ortholog set using MUSCLE [27].
5. Aligned_multiple_faa_concatenator.pl: Combines multiple sequence alignments generated in step 4, incorporating strings of "X" characters for ORFs not detected in particular taxa. Contains an option to exclude sequence columns containing data present only below some threshold value.

### Implementation

ORFcor is implemented in Perl (v5.10.1), multithreaded using the Perl “Parallel::ForkManager” module, and tested using Ubuntu Linux v10.04. The BLAST+ package [24] is a required dependency, and MUSCLE [27] and HMMER3 [28] are required for other pipeline steps. TRANSEQ in the EMBOSS [32] package is required to analyze nucleotide sequences. Using the ORFcor pipeline to construct and correct 31 ortholog sets from the 123 *Enterobacteriaceae* proteomes described above in “Test datasets and validation”, all according to default parameters using 16 AMD Opteron 618 processors (2.00 GHz), took a wall time of 6 min 21 sec, of which 1 min 41 sec comprised the ORFcor step, with a peak RAM usage of 832 MB. The version of ORFcor used in this manuscript is attached as File S2; see www.currielab.wisc.edu/downloads.html for the most recent version.

## Results

### Performance of ORFcor on Simulated Data

**Figure 2:**
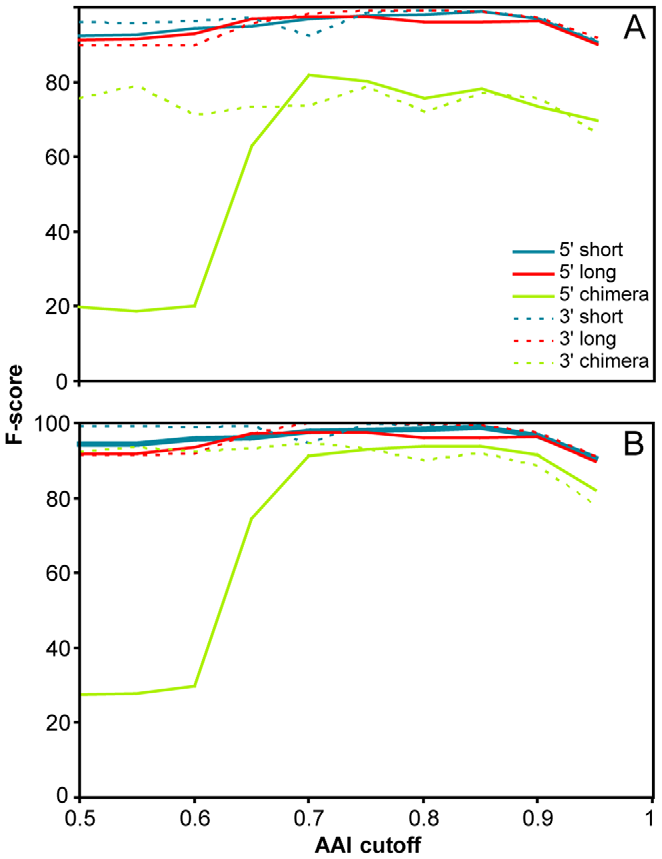
Effect of varying *d* (the minimum identity required between two protein sequences to be used for correction) on ORFcor performance measured using the F-score. Simulations using test datasets #1 and #2 are shown in panels A and B, respectively.

The performance of the ORFcor algorithm was examined using simulated data whereby ORF extensions, deletions, and chimeras were artificially introduced into otherwise high-quality sequences (see "Materials and Methods"). A wide range of algorithm parameters were examined on data having different ORF annotation inconsistency frequencies and sizes, and representative results are shown in Table 1 (see File S1 for complete results). Both sensitivity and specificity for correction of under- and overextensions approached 100%, with performance improving as the length of the simulated under- and overextensions increased. Chimera detection was similarly specific but less sensitive, likely due to BLAST inaccurately extending alignments into the chimeric region despite abundant mismatches, with sensitivity towards chimera detection improving substantially as simulated chimera lengths were increased. Increasing the minimum identity required for sequences to be used as references for correction (parameter *d*) resulted in a trade-off between correction accuracy and sensitivity, evident from the relatively constant F-scores shown in Figure 2, except for chimera correction below<*d* = 0.7 where false-positive corrections where common. Corrections made by ORFcor were also typically highly accurate (considering true-positive corrections), inserting or deleting the correct number of amino acids in the overwhelming majority of cases (Table 1). Interestingly, improved ORFcor sensitivity often led to decreased correction accuracy, suggesting the existence of sequences for which perfect correction is inherently difficult by this algorithm. Large miscorrections were occasionally observed during this simulation when an error was randomly incorporated into a reference sequence that was falsely corrected even without error incorporation; imperfect corrections otherwise differed from true lengths by only a few AA (Table 1).

### Performance of ORFcor on Complete and Enterobacteriaceae Proteomes

In reality, the test dataset used for the above simulations is optimistic because the sequences most likely to be falsely corrected (i.e., those without multiple closely related reference sequences in the same genus) were excluded during dataset construction, a necessity given the lack of an appropriate gold standard. Whereas the test dataset well-represents sets of closely related taxa, comparisons using more distantly related taxa and/or less conserved orthologs are likely to be less sensitive and specific. To explore this further, we obtained 5,575 proteins from the proteomes of 123 *Enterobacteriaceae* belonging to 31 ortholog families using the HMMs described in "Materials and Methods". Given that these 31 ortholog families were chosen based on their high conservation throughout all bacteria [25] and are therefore assumed to be present in each genome, this represents a 98.27% detection rate (5,575 proteins out of a possible 5,673). Interestingly, the proteins that were missed with the highest frequency (e.g., COG0099: 81.96% detection rate) were those having the shortest sequence lengths, thereby requiring the lowest e-value thresholds for the HMMs we used (1×10^−10^). Because e-values depend on sequence length, decreasing short sequence lengths lowers e-values more than proportionally identical decreases for longer sequences. This result highlights that sequence truncation (due to either start site prediction errors or low sequence quality) makes orthologs more difficult to detect using methods that yield e-values as results (e.g., BLAST or HMMs).

The results of ORFcor correction of the *Enterobacteriaceae* dataset are shown in Table 2. Using a minimum identity threshold *d* = 0.75 resulted in 146 true corrections (2.62% of the input dataset) compared with 35 false corrections (0.63% of the input dataset). Of these 35 false corrections, 30 were sequences belonging to COG0525 from *Yersinia* and its close relatives, which were longer than COG0525 sequences from other *Enterobacteriaceae*, but corrected because of a high confidence consensus start position inferred from the many other *Enterobacteriaceae* included using this relatively generous identity threshold. The other 5 false corrections were for highly diverged sequences belonging to endosymbiont taxa. The majority of true corrections occurred at 5′ sequence ends, and comprised 81 overextensions, 51 truncations, and 1 chimera (1.45%, 0.91% and, 0.02% of the total dataset, respectively). Interestingly, most of these corrections were restricted to a few ortholog sets (e.g., COG0048, COG0495 and COG0525) in which the conserved ATG start site was apparently replaced by an alternative start codon (GTG or TTG) that was not accommodated in their automated gene predictions. The existence of these errors was therefore conserved in related taxa but their start site locations were not, likely due to weaker selection on these upstream non-coding regions. True corrections at 3′ sequence ends were rare, comprising 4 overextensions, 8 truncations and 1 chimera (0.07%, 0.14%, and 0.02% of the total dataset, respectively). False corrections were made exclusively for 5′ overextensions except for one 5′ truncation. Overall, 4.17 X more true corrections were made compared to false corrections, indicating a modest improvement in data quality using a minimum identity threshold *d* = 0.75.

**Table 2:** Performance of ORFcor on 123 complete genome sequences belonging to the *Enterobacteriaceae* using default settings, except as indicated.

	Minimum identity threshold *d* = 0.75	Minimum identity threshold *d* = 0.90
*5′ Chimeras*		
True corrections (% total ORFs)	1 (0.02)	1 (0.02)
False corrections (% total ORFs)	0 (0.00)	0 (0.00)
*3′ Chimeras*		
True corrections (% total ORFs)	1 (0.02)	1 (0.02)
False corrections (% total ORFs)	0 (0.00)	0 (0.00)
*5′ Overextensions*		
True corrections (% total ORFs)	81 (1.45)	78 (1.40)
False corrections (% total ORFs)	34 (0.61)	0 (0.00)
*3′ Overextensions*		
True corrections (% total ORFs)	4 (0.07)	4 (0.07)
False corrections (% total ORFs)	0 (0.00)	0 (0.00)
*5′ Truncations*		
True corrections (% total ORFs)	51 (0.91)	50 (0.90)
False corrections (% total ORFs)	1 (0.02)	0 (0.00)
*3′ Truncations*		
True corrections (% total ORFs)	8 (0.14)	6 (0.11)
False corrections (% total ORFs)	0 (0.00)	0 (0.00)
*All error types*		
True corrections (% total ORFs)	146 (2.62)	140 (2.51)
False corrections (% total ORFs)	35 (0.63)	0 (0.00)

We also ran ORFcor on the same test dataset using an elevated minimum identity threshold *d* = 0.9 (Table 2). Using this more stringent threshold, we expected a decreased false-positive rate because: (i) most endosymbiont sequences would be excluded from correction due to their diverging from nearly all sequences to a degree beyond this threshold; and (ii) *Yersinia* and related genomes would only be compared with each other and not the rest of the *Enterobacteriaceae* from which an incorrect consensus was derived and therefore not falsely corrected. Indeed, these more stringent parameters eliminated all false corrections. As expected from the experiments using simulated data (Table 1 and Figure 2), sensitivity was slightly reduced using *d = *0.9 with 140 ORFs truly corrected (2.51% of the test dataset) compared to 146 truly corrected ORFs (2.62% of the test dataset) using *d* = 0.75. This equates to 95.89% of the corrections made using *d = *0.75 being maintained using *d* = 0.9.

### Evaluating the Stability of ORFcor Corrections in Increasingly Diverse Ortholog Sets

**Figure 3:**
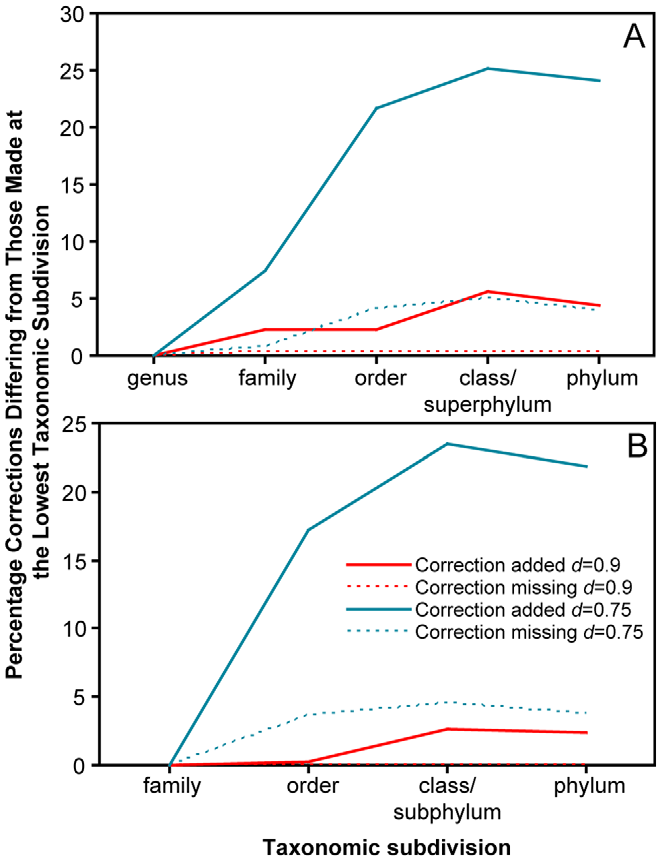
Stability of ORFcor corrections with increasing taxonomic divergence of the dataset. Values shown indicate ORFcor corrections that differ between test datasets subdivided at the lowest (genus in A; family in B) and higher taxonomic subdivisions. Genomes not classified at particular taxonomic levels were excluded from analysis.

We further characterized the stability of ORFcor corrections towards increasing diversity of the input ortholog sets using non-simulated data. All complete bacterial genomes were obtained from NCBI and subdivided according to their NCBI taxonomies at the level of genus, family, order, class/subphylum (class is otherwise undefined for Proteobacteria), and phylum. Each subdivision having ≥6 genomes (therefore meeting ORFcor default parameters) was then analyzed using ORFcor with *d* = 0.75 and *d* = 0.9, and the extent to which corrections at each taxonomic level differed from those made for data subdivided at the genus and family levels was determined (Figures 3A and 3B, respectively), considering only taxa classified at both taxonomic levels. The rationale for this experiment was that corrections made at lower taxonomic levels would be free from false correction due to the presence of more numerous, divergent taxa (e.g., as for *Yersinia* and relatives corrected using *d* = 0.75 in the analysis of *Enterobacteriaceae* described above). As expected, more sequences were corrected using *d* = 0.75 (610 when subdivided by genus, i.e., 2.56% of the entire dataset; 900 when subdivided by family, i.e., 2.84% of the entire dataset) than using *d* = 0.9 (345 when subdivided by genus, i.e., 1.45% of the entire dataset; 420 when subdivided by family, i.e., 1.33% of the entire dataset). Corrections made using *d = *0.9 changed very little between taxonomic subsets, confirming the high specificity of this threshold suggested by the previous analyses (Tables 1 and 2). In contrast, the number of corrections made using *d = *0.75 increased dramatically when using broader taxonomic levels, likely due to the lack of homologous start and stop sites at these taxonomic levels and sequence similarities. Based on these results, we conservatively recommend the routine use of *d = *0.9 and have set this as the ORFcor default. However, we also suggest that using lower *d* values combined with subdivision of the dataset (e.g., based on taxonomy at the genus level) may be an effective method of increasing the sensitivity of ORFcor without unduly sacrificing specificity. Which method is most effective should be evaluated on a case-by-case basis by examination of the ORFcor summary output files or alignments and the corrected sequences.

## Discussion

In this work, we present ORFcor as a method to correct inconsistencies in pre-existing sequence data resulting from inconsistent annotations of ORF start and stop sites and ORF chimerism. All of these inconsistency types were observed at both 5′ and 3′ sequence ends in our manual evaluation of 31 conserved proteins extracted from 123 complete *Enterobacteriaceae* genomes (Table 2) at a frequency of ∼2.62% per protein, a rate consistent with those obtained during our further analysis of all complete bacterial genomes using various ORFcor parameters. This rate of ORF annotation inconsistencies is likely an underestimate given the slow evolutionary rate of many of the proteins used in our dataset, and would be higher still if draft-quality genomes were also included. Genuine start and stop site prediction errors can result from the imprecision of the automated computational methods used, sequencing errors introducing premature stop codons or masking start site signatures, and draft quality genome sequencing truncating ORFs that run over contig edges. Although some ORF annotation differences are undoubtedly genuine, differentiating these from sequencing errors is essentially impossible without experimental validation and its incorporation into genome annotations (e.g., using Gene Ontology-style evidence codes [33]). Furthermore, we argue that the preservation of such diversity is not always desirable from a phylogenomic perspective, e.g., for reversible programmed frameshifts or where only the shorter of multiple genuine start sites is specified.

In our data, inconsistencies in 3′ sequence ends were much rarer than those at 5′ ends, likely due to the relative simplicity of finding the first stop codon along an ORF, compared to the greater difficulty in discriminating between multiple potential start codons at the 5′ sequence end. Unusually short 3′ sequence lengths may correspond to sequencing errors that introduce a premature stop codon, or programmed frameshifts within these ORFs. We also observed that 3′ sequence ends were often more heterogeneous than 5′ ends, necessitating more stringent thresholds for making these corrections. In contrast, our analyses suggest that inconsistencies at 5′ sequence ends are relatively common, especially where alternative start codons like GTG and TTG occur. Whereas these inconsistencies may reflect bona fide alternative start sites, the lack of ATG codons at conserved positions preceding these alternative potential start codons argues for their artificiality. The existence of non-conserved ATG codons following conserved start sites similarly argues for the artificiality of these apparent ORF truncations. The decreased detection of short orthologs using our HMM-based pipeline also highlights the increased difficulty in recognizing short error-containing ORFs for further analysis, although this was not a focus of this study.

There is clearly a tradeoff between the sensitivity and specificity of ORFcor, especially governed by the minimum identity threshold *d* which defines the similarity of sequences used to define consensus start and stop sites. Whereas using a high identity threshold can restrict the number of sequences required for comparison such that the minimum number of reference sequences used for correction is not met, using a lower identity threshold may allow inclusion of reference sequences having start and/or stop positions that are not homologous to the true start and/or stop positions of the query sequence and can result in excessively aggressive corrections due to biased oversampling of divergent reference sequences (e.g., as described for *Yersinia* COG0525 in the *Enterobacteriaceae* correction experiment). Whereas we have set a high identity threshold *d* = 0.9 as a conservative default (Figure 3), using a lower threshold may sometimes be more appropriate, e.g., when ortholog sets are taxonomically restricted or less well-conserved than the sequences in our test dataset.

Parameters aside, it is important to ensure that the input data used as input for ORFcor do not violate the central assumption of the method, namely, that each set of input sequences has conserved start and stop sites and are sufficiently closely related such that deviant sequences can be reliably recognized and corrected. A related assumption is that all sequences evolve approximately equally across their entire sequence length, because whereas the features of interest to ORFcor (i.e., the start and stop sites) are located at the sequence ends, calculation of homology (e.g., by BLAST and HMMs) is conducted across the entire sequence length. Both of these assumptions appear to be violated by sequences from obligate endosymbiont genomes in the *Enterobacteriaceae* dataset. Many sequences from these taxa lacked homologous sister sequences with conserved stop and start sites due to their accelerated rate of evolution [29], making their correction by ORFcor error-prone and insensitive. Exclusion of such evolutionarily-anomalous sequences prior to analysis is therefore advisable to meet the assumptions of the ORFcor algorithm. The need to specifically account for such biases during phylogenetic analysis has also been noted elsewhere (e.g., [34], [35]).

In summary, we highlight that ORF structural inconsistencies are commonplace among publicly available genomic data. Given its excellent performance on test and real data, approaching 100% accuracy after accounting for sequences with evolutionary histories incompatible with the assumptions of the algorithm, we suggest that ORFcor effectively improves such data and thereby represents a useful method to improve analyses in which genes are compared to each other, especially at the whole-genome scale using the phylogenomic approach [1], [2]. Our method is highly modular and can be easily combined with other analyses as desired (e.g., to correct frameshifts, which ORFcor does not attempt due to working at the protein level and its inability to differentiate between programmed frameshifts and those introduced by sequencing error). Whereas some authors have argued in a phylogenetic context that imperfect data should be omitted due to their confounding influence [36], such deficiencies are often less than the value added by such imperfect sequences through increased taxon sampling [37] and are further ameliorated in the phylogenomic approach by the ample phylogenetic signal provided by many genes [38]. The effect of similar biases on comparative methods that are not explicitly phylogenetic have, to our knowledge, not been explicitly studied, but might be expected to be detrimental in cases where truncation reduces the length of an ORF such that homology cannot be accurately determined due to insufficient statistical signal. Accommodating such biases (e.g., using ORF fragment linkage [8]) remains an important topic for future research.

## Supporting Information

File S1 **Complete results for ORFcor run under various parameter settings.** Sensitivity and specificity are calculated for each inconsistency type as the union of the entire 50 replicate datasets and also as the mean and standard deviation for each set of replicates. The mean and standard deviation for the number of corrections and the mean length of deviation from the true corrected length for both corrected (mismatches) and all sequences (accuracy) are given for each error type.
(OUT)

File S2 **The complete ORFcor software package as used in this research.** For the most recent version, see: currielab.wisc.edu/downloads.html.
(GZ) Click here for additional data file.
